# Pancreatic islet expression profiling in diabetes-prone C57BLKS/J mice reveals transcriptional differences contributed by DBA loci, including *Plagl1 *and *Nnt*

**DOI:** 10.1186/1755-8417-2-1

**Published:** 2009-01-22

**Authors:** Abraham A Anderson, Joan Helmering, Todd Juan, Chi-Ming Li, Jocelyn McCormick, Melissa Graham, Daniel M Baker, Michael A Damore, Murielle M Véniant, David J Lloyd

**Affiliations:** 1 Department of Computational Biology, Amgen Inc., One Amgen Center Dr, Thousand Oaks, CA 91320, USA; 2 Department of Metabolic Disorders, Amgen Inc., One Amgen Center Dr, Thousand Oaks, CA 91320, USA; 3 Department of Protein Sciences, Amgen Inc., One Amgen Center Dr, Thousand Oaks, CA 91320, USA; 4 Department of Molecular Sciences, Amgen Inc., One Amgen Center Dr, Thousand Oaks, CA 91320, USA

## Abstract

**Background:**

C57BLKS/J (BLKS) mice are susceptible to islet exhaustion in insulin-resistant states as compared with C57BL6/J (B6) mice, as observed by the presence of the leptin receptor (*Lepr*) allele, *Lepr*^db/db^. Furthermore, DBA2/J (DBA) mice are also susceptible to β-cell failure and share 25% of their genome with BLKS; thus the DBA genome may contribute to β-cell dysfunction in BLKS mice.

**Results:**

Here we show that BLKS mice exhibit elevated insulin secretion, as evidenced by improved glucose tolerance and increased islet insulin secretion compared with B6 mice, and describe interstrain transcriptional differences in glucose response. Transcriptional differences between BLKS and B6 mice were identified by expression profiling of isolated islets from both strains. Genomic mapping of gene expression differences demonstrated a significant association of expression differences with DBA loci in BLKS mice (*P *= 4×10^-27^).

**Conclusion:**

Two genes, *Nicotinamide nucleotide transhydrogenase *(*Nnt*) and *Pleiomorphic adenoma gene like 1 *(*Plagl1*), were 4 and 7.2-fold higher respectively in BLKS islets, and may be major contributors to increased insulin secretion by BLKS islets. Contrary to reports for B6 mice, BLKS mice do not harbor a mutant *Nnt *gene. We detected 16 synonymous polymorphisms and a two-amino acid deletion in the *Plagl1 *gene in BLKS mice. Several inflammatory glucose-responsive genes are expressed at a higher level in BLKS, suggesting an inflammatory component to BLKS islet dysfunction. This study describes physiological differences between BLKS and B6 mice, and provides evidence for a causative role of the DBA genome in β-cell dysfunction in BLKS mice.

## Background

Type 2 diabetes mellitus can be considered as a two-stage disease. First the body becomes resistant to circulating insulin, and second, when coupled with pancreatic β-cell dysfunction, overt diabetes precipitates [1]. Insulin resistance alone, without β-cell exhaustion, will not lead to hyperglycemia. In this case the islet compensates by increasing insulin production and β-cell populations are maintained. Differences in β-cell adaptation to increased insulin demand likely reflect an underlying genetic component. Indeed, in B6 mice, but not in genetically dissimilar BTBR mice, insulin resistance induced by the leptin *ob *allele is well compensated by islet expansion [2]. The genetic alteration in BTBR mice which underpins this failure to adapt to increased insulin requirements was recently identified and localized to a gene contributing to islet vascularization [3].

Obese (*Lepr*^db/db^) B6 and 129/J mice are insulin resistant but compensate this potentially pathogenic process by islet hypertrophy and hyperinsulinemia [4,5]. However, obese (*Lepr*^db/db^) DBA mice not only develop insulin resistance but also severe diabetes due to β-cell loss. Thus the DBA background may be considered a diabetogenic strain exhibiting a predisposition to β-cell failure. DBA mice copiously secrete insulin (10-fold over basal) in response to a glucose bolus compared with a modest (3-fold) increase in B6 mice [6]. This hypersecretion phenotype (here after referred to as elevated insulin secretion) is retained in isolated islets, and confirms a functional difference between these two strains specific to the islet. These data suggest that elevated secretion of insulin may be deleterious to the β cell and results in β-cell exhaustion in DBA mice.

BLKS mice also exhibit pancreatic islet failure when made insulin resistant by the *ob *or *db Lepr *alleles [5,7,8], as evidenced by gross islet atrophy and β-cell loss. This strain is closely related to B6; however, it harbors a small contribution from the DBA strain. Genetic analysis of the BLKS mice has uncovered a contribution of approximately 71% from B6, 25% from DBA, and 4% from other strains in BLKS mice [9-11]. Given the clear differences in severity of diabetes between B6-*Lepr*^db/db ^mice and BLKS-*Lepr*^db/db ^mice, and the similarity between BLKS and DBA mice, it is tempting to speculate that islet sensitivity may be conferred by alleles from the DBA genome.

In this report we investigated islet function in non-obese B6 and BLKS mice. We found that BLKS mice are profoundly more glucose tolerant than B6 and this difference was associated with elevated secretion of insulin in islets. We investigated the differences in gene expression in isolated islets between the two strains, in both basal and stimulated (insulin secretion stimulated) conditions, and found that 75% of the gene expression differences in the BLKS islets were contributed from genes within DBA loci. Further, several genes regulated by glucose in both strains suggest early inflammatory effects associated with increased glucose (*Txnip*,*Lnc2*,*Gad1 Slc7a3 *and *Spp1*). Notably, we identified significantly higher expression of *Nnt *and *Plagl1 *(ZAC1) in BLKS islets, two genes previously associated with islet dysfunction.

## Results and discussion

### Glucose homeostasis in non-obese BLKS and B6 mice

**Figure 1:**
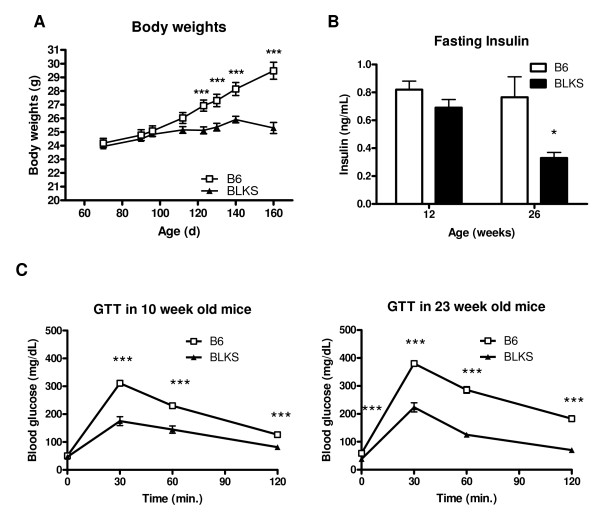
**Glucose homeostasis in B6 and BLKS mice**. (A) Body weights of B6 and BLKS male mice (*n *= 13 for each strain) were monitored from 10 weeks to 26 weeks of age. (B) Fasting (4 h) plasma insulin was analyzed in 10-week (*n *= 13) and 23-week (*n *= 8) -old B6 and BLKS male mice. (C) Glucose tolerance was assessed in 10 and 23-week-old B6 and BLKS male mice, following an intraperitoneal injection of glucose (2 g/kg). All data represent means and s.e.m. and tested using a two-tailed Student's *T*-test assuming unequal variances; * *P *< 0.05, ** *P *< 0.01, *** *P *< 0.001.

Although glucose homeostasis has been extensively studied in BLKS-*Lep*^db/db ^mice, few reports have shown clear differences in non-obese BLKS mice compared with B6 mice. To better understand the differences between both these strains, we analyzed fasting glucose in young (12 weeks old) and aged (26 weeks old) mice. At 12 weeks of age, fasting glucose levels between B6 and BLKS mice were similar (113.3 ± 5.7 mg/dL *vs. *100.7 ± 3.6 mg/dL, respectively) as well as at 26 weeks (118.4 ± 4.2 mg/dL *vs. *111.0 ± 17 mg/dL). Interestingly, B6 mice and BLKS mice have similar body weights at younger ages but after 16 weeks of age the B6 mice continue to gain weight whereas BLKS mice do not (Figure 1A). Also, in 12-week-old-mice insulin levels were the same in the two strains (Figure 1B). However, at 26 weeks B6 mice had significantly higher insulin levels (*P *= 0.018) than BLKS mice, and could reflect the difference in body weights. We found no differences in glucagon levels (data not shown). The difference in insulin levels in older mice may also represent a difference in glucose homeostasis. To investigate this further, glucose tolerance tests (GTTs) were performed at 10 and 23 weeks of age (Figure 1B). At both ages, glucose tolerance between B6 and BLKS mice was clearly different. Although 10-week-old B6 and BLKS mice had similar basal fasting blood glucose levels, at 30, 60 and 120 min after an intraperitoneal injection of glucose B6 mice were less glucose tolerant than the BLKS mice. Similar results were obtained at 23 weeks of age; however, at this age the basal fasted blood glucose values for the BLKS mice were statistically lower than the B6 mice. Both lines of mice were more glucose intolerant at 23 weeks than at 10 weeks of age (blood glucose spike at 30 min was increased in both strains). The glucose intolerance observed in the B6 mice at 23 weeks could be related to the higher body weight and some degree of peripheral of insulin resistance. On the other hand, the younger B6 and BLKS 10-week-old mice had similar body weights yet clearly were dissimilar in glucose tolerance; furthermore, a report by Goren et al [12] illustrated that 8 to 10-week-old B6 and BLKS mice are very similar when challenged in an insulin tolerance test. Taken together, we interpret these data to reflect a pancreatic difference between these two strains to account for the differences in glucose tolerance.

### Islet insulin secretion and pancreatic immunohistochemistry

**Figure 2:**
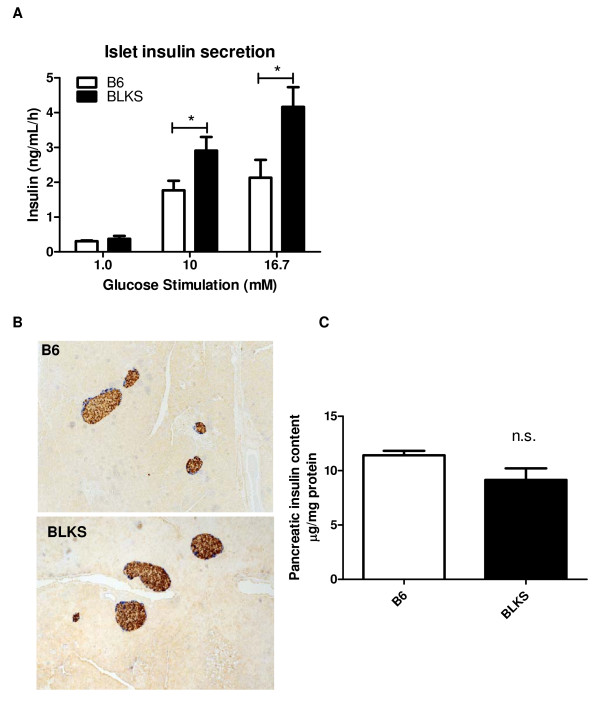
**Insulin secretion is higher in BLKS mice than B6 mice**. (A) Glucose-stimulated insulin secretion was investigated in isolated islets from 12-week-old B6 and BLKS male mice. Following 48 h recovery post-isolation, the islets were stimulated with 2, 10 or 16.7 mM glucose for 1 h. Insulin release into the media was monitored. (B) Immunohistochemistry was performed in pancreatic islets from 26-week-old male mice revealing no gross differences in islet appearance or size. Insulin (oxidized DAB-brown) and glucagon (Vector Blue product) expressing cells are shown. (C) Pancreatic insulin content was assessed in pancreata excised from 15-week-old male B6 and BLKS mice (*n *= 3/group). No statistically significant differences were detected. All data represent means and s.e.m. and tested using a two-tailed Student's *T*-test assuming unequal variances; * *P *< 0.05; *n.s*. not significant.

To investigate if the enhanced glucose clearance in BLKS mice was attributed to increased insulin secretion, glucose-stimulated insulin secretion was measured in isolated islets from both B6 and BLKS mice (Figure 2A). Islets of BLKS mice secreted significantly more insulin than B6 mice at the higher glucose concentrations tested, providing further support for an elevated secretion phenotype. These data have been shown for DBA mice [6,13], but to date have not been reported for BLKS mice.

**Figure 3:**
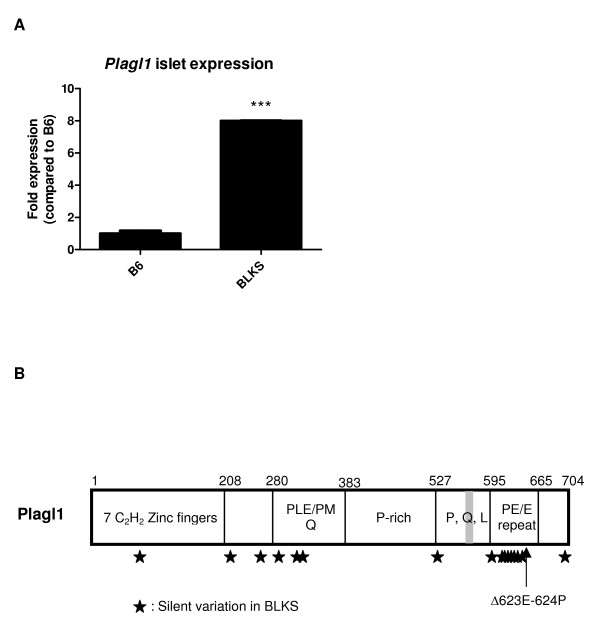
***Plagl1 *expression and genetic differences in BLKS mice**. (A) Quantitative RT-PCR analysis of the *Plagl1t *RNA in B6 and BLKS in isolated islet cultured in high glucose conditions. Data represent means and standard deviations of multiple mice (B6 *n *= 8; BLKS *n *= 4) and tested using a two-tailed Student's *T*-test assuming unequal variances; *** *P *< 0.001. (B) Diagram of the polypeptide structure of Plagl1 identifying the functional domains. Numbers refer to the mouse amino-acid positions, and letters refer to amino acids. The genetic differences between B6 and BLKS genes are shown by the stars; the two-amino acid deletion in BLKS is shown by the arrow.

To determine if the difference in insulin secretion correlated with differences in β-cell mass, we performed histology and immunohistochemistry on pancreatic sections from both strains. No gross differences in islet number or appearance were observed in either B6 or BLKS mice as assessed in multiple mice of each strain. Furthermore, following staining for insulin and glucagon, a similar composition of α and β cells within the islet was found (Figure 2B). Thus the difference in insulin secretion was not associated with any qualitative difference in β-cell mass. To investigate if any quantitative differences in β-cell mass were present between the two strains, we analyzed pancreatic insulin content (Figure 3C). Pancreata from BLKS mice were found to have a small but non-significant reduction in pancreatic insulin content compared with B6 mice. This, however, is unlikely to explain the elevated insulin secretion observed in the BLKS mice.

### Islet gene expression differences between glucose concentrations

Given that islet dysfunction is a characteristic of BLKS-*Lepr*^db/db ^mice but not of B6-*Lepr*^db/db ^mice, and is associated with heightened insulin secretion in non-obese BLKS mice, we hypothesized that these physiological outcomes may be reflected in differences in islet gene expression. We investigated gene expression in normal (non-diabetic) mice with the intention of analyzing expression in islets with similar compositions of α, β, and δ cells. Furthermore, we speculated differences in gene expression may only become apparent after a period of extended secretion, which would expose genes involved in islet dysfunction/adaptation. To this end, islets were isolated from 10 B6 and 10 BLKS mice, and cultured for 2 days in 11 mM glucose (each individual mouse's islets were cultured separately). After this period, islets from five B6 mice were cultured in 5 mM glucose and from five B6 mice in 20 mM glucose for an additional 24 h. BLKS islets were treated similarly. RNA isolated from each individual mouse's islets was used to investigate global changes in gene expression between the strains in low and high-glucose conditions. We assessed the common differences due to glucose concentration (Table 1), and differences between strains (Table 2).

**Table 1:** Gene expression fold-change differences in islets from low to high glucose in BLKS and B6 mice

Probe	Gene	Description	Interstrain fold difference (BLKS/B6)		Glucose response (fold change)	
			
			Low Gluc.	High Gluc.	B6	BLKS
A_51_P402943	*S100A9*	S100 calcium-binding protein A9	1.21	3.28	1.59	4.31

A_51_P438805	*TXNIP*	thioredoxin-interacting protein	-1.72	-2.01	4.16	3.56

A_51_P358765	*SPP1*	secreted phosphoprotein 1	2.46	3.76	2.12	3.24

A_51_P275016	*SLC7A3*	solute carrier family 7 member 3	1.49	1.97	2.13	2.82

A_51_P372550	*CGREF1*	cell growth regulator with EF-hand domain 1	1.04	1.65	1.76	2.79

A_51_P510156	*LCN2*	lipocalin 2	1.98	2.07	2.31	2.41

A_52_P94482	*TTYH1*	tweety homolog 1	1.73	1.84	2.23	2.37

A_51_P194149	*TTR*	transthyretin	-1.31	1.13	1.59	2.37

A_51_P428384	*TTYH1*	tweety homolog 1	1.58	2.19	1.70	2.36

A_52_P326399	*FKBP11*	FK506-binding protein 11	1.50	1.48	2.39	2.36

A_51_P130727	*FKBP11*	FK506-binding protein 11	1.65	1.64	2.34	2.33

A_51_P227275	*CSN3*	casein kappa	1.43	1.54	2.15	2.31

A_51_P370458	*A030006P16RIK*	RIKEN cDNA	1.01	1.64	1.41	2.29

A_51_P391955	*2310032F03RIK*	RIKEN cDNA	1.10	1.21	2.07	2.27

A_51_P346938	*LRG1*	leucine-rich alpha-2-glycoprotein 1	1.18	1.17	2.22	2.20

A_52_P187058	*NPTX2*	neuronal pentraxin II	1.67	2.38	1.51	2.16

A_51_P435911	*HSD3B7*	hydroxy-delta-5-steroid dehydrogenase, 3 beta- and steroid delta-isomerase 7	1.17	1.69	1.46	2.11

A_51_P162955	*SERPINA7*	serpin peptidase inhibitor member 7	-1.01	1.21	1.69	2.07

A_52_P547328	*TRAM1*	translocation-associated membrane protein 1	1.09	1.51	1.49	2.06

A_51_P272876	*FAM46A*	family with sequence similarity 46, member A	1.59	1.72	1.88	2.03

A_52_P144310	*GAD1*	glutamate decarboxylase 1	1.02	-1.23	-1.63	-2.04

A_51_P199352	*2310015B20RIK*	RIKEN cDNA	-1.25	-1.71	-1.62	-2.21

Genes are sorted for the subset with BLKS glucose response (fold change) >2 (*p *< 0.001, Benjamini-Hochberg FDR 0.8).

**Table 2:** Gene expression fold-change differences in islets from BLKS mice compared with those from B6 mice

			Interstrain fold difference (BLKS/B6)				
						
Probe	Gene	Description	Low Gluc.	High Gluc.	Chromosome	Mb	Genetic Origin
A_51_P417251	*6330403K07RIK*	RIKEN cDNA	4.32	17.43	11	71.1	DBA

A_52_P532456	*PLAGL1*	pleiomorphic adenoma gene-like 1	2.93	7.15	10	12.8	DBA

A_51_P113395	*NNT*	nicotinamide nucleotide transhydrogenase	1.94	4.22	13	116.6	DBA

A_52_P395228	*NNT*	nicotinamide nucleotide transhydrogenase	2.01	4.07	13	116.6	DBA

A_51_P113399	*NNT*	nicotinamide nucleotide transhydrogenase	1.79	3.85	13	116.6	DBA

A_51_P422030	*FLJ22709*	hypothetical protein	1.99	3.82	8	69.8	DBA

A_51_P358765	*SPP1*	secreted phosphoprotein 1	2.46	3.76	5	103.6	B6

A_51_P253984	*PCP4*	Purkinje cell protein 4	1.93	3.52	16	96.9	DBA

A_51_P402943	*S100A9*	S100 calcium-binding protein A9	1.21	3.28	3	90.8	DBA

A_52_P542860	*BTBD9*	BTB domain-containing 9	1.99	3.27	17	28.7	DBA

A_51_P440682	*CAP1*	CAP, adenylate cyclase-associated protein 1	1.82	3.17	4	122.3	DBA

A_52_P3029	*AGPAT4*	1-acylglycerol-3-phosphate O-acyltransferase 4	1.42	2.92	17	10.9	DBA

A_51_P323620	*THYN1*	thymocyte nuclear protein 1	1.74	2.91	9	26.9	other

A_51_P346165	*AGPAT4*	1-acylglycerol-3-phosphate O-acyltransferase 4	1.42	2.89	17	10.9	DBA

A_51_P118417	*SCPEP1*	serine carboxypeptidase 1	1.70	2.87	11	89.0	DBA

A_51_P454873	*NPY*	neuropeptide Y	1.97	2.72	6	49.8	B6

A_51_P491017	*GNPTG*	N-acetylglucosamine-1-phosphate transferase, gamma subunit	1.82	2.71	17	23.4	DBA

A_52_P748882	*ENO2*	enolase 2	1.62	2.58	6	124.8	DBA

A_51_P167535	*FABP3*	fatty acid-binding protein 3	1.88	2.53	4	129.8	DBA?

A_51_P130028	*ENO2*	enolase 2	1.63	2.53	6	124.8	DBA

A_51_P101545	*HGFAC*	HGF activator	-1.92	-2.54	5	34.1	DBA

A_51_P337269	*ALDOB*	aldolase B, fructose-bisphosphate	-1.17	-2.66	4	49.6	B6

A_51_P215627	*PLAC9*	placenta-specific 9	-1.89	-2.67	14	23.6	DBA

A_52_P644774	*ZZEF1*	zinc finger, ZZ-type with EF-hand domain 1	-1.92	-2.73	11	73.0	DBA

A_51_P517075	*SERPINF1*	serpin peptidase inhibitor, clade F member 1	-1.50	-2.78	11	75.5	DBA

A_52_P213889	*TMC7*	transmembrane channel-like 7	-1.87	-2.83	7	114.5	DBA

A_52_P613498	*4833420G17RIK*	RIKEN cDNA	-2.04	-3.01	13	116.7	DBA

A_51_P376656	*SYNPR*	synaptoporin	-2.13	-3.08	14	11.4	DBA

A_52_P50496	*HLA-A*	major histocompatibility complex, class I, A	-2.23	-3.16	17	32.1	DBA

A_51_P509961	*SLC5A10*	solute carrier family 5 member 10	-1.21	-3.24	11	61.8	DBA

A_51_P246677	*REC8L1*	REC8-like 1	-2.28	-3.61	14	50.5	DBA

A_51_P392518	*THUMPD1*	THUMP domain-containing 1	-2.83	-4.30	7	115.6	DBA

A_51_P146560	*MSLN*	mesothelin	-2.31	-4.36	17	23.9	DBA

A_52_P137500	*AK046255*	Unknown cDNA	-2.70	-5.06	X	131.8	DBA

A_51_P443443	*5830417I10RIK*	RIKEN cDNA	-2.75	-5.46	3	89.0	DBA

A_52_P231729	*H2-Q1*	major histocompatibility complex Q1b	-8.74	-29.31	17	33.4	DBA

Shown is the subset with fold change >2.5 under high glucose conditions (*p *< 0.001, Benjamini-Hochberg FDR 0.8). Genes are sorted by the interstrain fold difference in high glucose conditions.

Table 1 shows gene expression fold-change differences between low and high-glucose conditions in BLKS and B6 mice. We highlight the subset with BLKS glucose response of at least 2-fold. These transcriptional differences associated with glucose response in islets suggest an underlying inflammatory component. *Lipocalin 2 *(*Lcn2*) transcription increases 2.4-fold in response to higher glucose levels and is 2.07-fold higher in BLKS islets. This gene is regulated by the cytokine leptin in insulinoma cells [14] and interestingly BLKS mice also have 40% more of a specific leptin receptor (*Leprotl1*) at high glucose levels – 1.44 and 1.09-fold under high and low-glucose conditions, respectively. *Lcn2 *is also known to be up-regulated in rat models of type 2 diabetes, and associated with inflammatory functions [15] including apoptosis. The glucose-responsive gene *Glutamate decarboxylase 1 (Gad1)*, which is an islet-derived autoantigen in insulin-dependent diabetes [16], may be a marker for β-cell loss or toxicity and has been shown to be glucose responsive. We observed a *Gad1 *decrease in both mouse models, but more so in the BLKS mice. The gene with the largest change due to glucose in BLKS mice was *S100a9*. This gene is expressed in monocytes and pancreatic cell lines, and is essential for pancreatic leukocyte infiltration [17,18]. In addition to being an inflammatory marker *S100a9 *is associated with islet autoimmunity [19]. Another glucose-responsive gene, *Slc7a3*, is likely an indirect marker for the presence of resident islet inflammatory cells. *Slc7a3 *is a polar amino acid transporter, which provides L-arginine that can be used in nitric oxide (NO) production. NO synthesis and overproduction has been associated with β-cell dysfunction in diabetic rats [20].

In addition to the inflammation-associated genes we also observed strong induction by glucose of the gene encoding *Thioredoxin interacting protein *(*Txnip*) in both BLKS and B6 mice. *Txnip *has been recently linked with glucose-induced β-cell loss [21]. Chen et al showed that glucose incubation up-regulated *Txnip *and increased apoptosis, which they speculated may be via the intrinsic mitochondrial apoptotic pathway. They also showed that constitutive over-expression is not necessary for apoptosis, but that the glucose-induced increase is sufficient. Furthermore, Parikh et al have shown that, in humans, *Txnip *over-expression reduces basal and insulin-stimulated glucose uptake [22], is elevated by glucose and suppressed by insulin. We confirm the increase of *Txnip *expression in response to glucose, although we do not observe any differential response to glucose between B6 and BLKS mice. On the contrary, BLKS mice have 2-fold lower levels of *Txnip *than B6 mice. Thus *Txnip *is unlikely to underlie the islet failure in BLKS mice, but more likely to represent the typical response to glucose.

### Islet gene expression differences between B6 and BLKS strains

Table 2 shows a list of genes which were more than 2.5-fold different in BLKS islets compared with B6 islets in high glucose conditions (column 4, High Gluc.). All members of this list were significantly different between strains, regardless of glucose concentration, after controlling the false discovery rate at 0.80 [23]. In addition to its induction by glucose within each strain (Table 1) *Spp1 *expression is also significantly different between strains and is 3.5-fold higher in BLKS islets (Table 2). *Spp1 *or osteopontin is a multifunctional cytokine and a potential diagnostic predictor of diabetic end-stage renal disease [24]. Arafat et al have shown that *Spp1 *averts cytokine-mediated β-cell toxicity through negative regulation of NO production [25]. As we have shown, both BLKS and B6 mice up-regulate *Slc7a3*, a gene associated with NO production, but BLKS mice always express higher levels (1.49 to 1.97-fold). Thus, the excess capacity for NO production in BLKS mice might counter the protective up-regulation of *Spp1*. Other genes such as *S100a9*, *Aldob*, and *Slc5a10 *demonstrate a stronger glucose response in only one of the mouse strains. S100a9, a diabetes-associated pro-inflammatory molecule [26], had a much larger increase due to increasing glucose in BLKS mice (4.3-fold) *vs. *B6 mice (1.6-fold). *Aldob*, encoding a glycolytic enzyme that is decreased in human diabetic islets [27], and *Slc5a10*, a sodium/glucose co-transporter, had stronger induction due to increasing glucose in B6 mice (1.5 and 3.5-fold respectively) *vs. *BLKS mice (1.1 and 1.6-fold respectively).

From Table 2, 32 genes were more than 2.5-fold different in expression levels in BLKS islets compared with B6 islets; 16 genes were expressed at higher levels and 16 genes expressed at lower levels. Thirty of these genes displayed fold differences with magnitudes between 2.5 and 7.15. However, two genes had notable differences above this range.

The first, an uncharacterized transcript (*6330403K07RIK*), was 17.4-fold higher in BLKS islets. At this time, this gene's product has no assigned function and has no known homology to any protein. Interestingly, two protein sequences were identified from the NCBI gene database corresponding to *6330403K07RIK *which had been translated from DBA genomic sequence and B6 mRNA sequence and were found to be different. The protein sequence from DBA mice (AAR87485) was different at four residues compared with the B6 sequence (BAB31072) over the 121 residues.

The second gene with profound differential expression was *H2-Q1*, with a 29.3-fold lower level in the islets from BLKS mice. *H2-Q1 *localizes to the H2 locus in mice on chromosome 17. B6 mice are known to be isogenic for the H2b haplotype and BLKS mice are a recombinant congenic strain carrying the H2d haplotype [28]. The difference in *H2-Q1 *expression likely represents a difference conferred by the two haplotypes, and the *H2-Q1 *gene is possibly not expressed in mice with the H2d haplotype. Furthermore, another gene from the H2 locus was identified in Table 2 (*H2-K1*), and suggests that the influence of H2 haplotype can manifest in multiple gene expression differences.

In further studies, we focused on the two genes *Plagl1 *and *Nnt*, which have previously been implicated in islet dysfunction in both humans and mice.

### Transcriptional differences in *Plagl1 *and *Nnt*

The second-most highly expressed gene in BLKS islets compared with B6 islets was *Plagl1 *(7.2-fold higher expression; Table 2). The expression state and magnitude was confirmed by qRT-PCR under the same conditions of high glucose in a separate experiment (Figure 3A). *Plagl1 *has been previously implicated in human transient neonatal diabetes mellitus (TNDM) [29], and can result from paternal uniparental disomy of chromosome 6, or paternal duplication of 6q24 (the TNDM locus). These genetic aberrations suggest the disease results from over-expression of an imprinted gene [29]. If BLKS islets are indeed predisposed to failure (when confronted with insulin resistance), then over-expression of *Plagl1 *would be consistent with the expression observed in human TNDM. Similarly, mice which over-express the human TNDM locus (including *Plagl1*) exhibit impaired glucose homeostasis [30]. Several functions for *Plagl1 *have been reported [31]. Interestingly, evidence that *Plagl1 *can regulate apoptosis may be relevant to the β-cell dysfunction in these mice. In BLKS mice, the *Plagl1 *gene is within a locus predicted to be inherited from the DBA parental strain. To investigate the underlying cause for the difference in *Plagl1 *expression we sequenced the four exons of the gene. Contrary to the published DNA sequence of *Plagl1 *in B6 mice, BLKS mice have 16 synonymous polymorphisms and a two-amino acid deletion (623Glu-624Pro; Figure 3B). These polymorphisms are contained within the third exon of the gene. The 16 synonymous polymorphisms may not affect the function of the protein, but could perhaps contribute to the higher expression of the *Plagl1 *transcript by affecting RNA stability. Additionally the 6 bp deletion could alter the RNA stability or possibly change the function of the protein product. The 623Glu-624Pro deletion in BLKS mice lies at the end of a repetitive Pro-Glu-Gln repeat region not found in the human paralog. Despite this structural difference within the *Plagl1 *gene of human and mice, functional differences have not been observed [31]. Thus it is uncertain whether the deletion of the 623Glu-624Pro is functionally relevant to the β-cell phenotype in BLKS mice.

We observed three different probes for *Nnt *that all showed a clear difference in expression (Table 2). On average, the *Nnt *was expressed at 4-fold higher levels in BLKS islets in high-glucose conditions. It was observed that the differences in *Nnt *expression were present in islets cultured in low-glucose conditions (Table 2, column 4); however, this was greater in islets following a stimulated pretreatment. B6 mice were recently shown to be glucose intolerant, as a result of impaired insulin secretion, and this was linked to mutation of the *Nnt *gene [32]. In this study, a quantitative trait locus (QTL) analysis in B6 and C3H/HeH mice revealed that expression of *Nnt *was 5-fold lower in B6 islets, and is similar with the 4-fold level we observed compared with BLKS islets. Nnt is a nuclear-encoded mitochondrial protein involved in detoxification of reactive oxygen species [33], which is crucial for their removal and to reduce their deleterious effect on mitochondrial ATP production. Intact *Nnt *will likely lead to higher ATP levels and thus enhanced insulin secretion in the β cell. To confirm the role of *Nnt *in insulin secretion, B6 mice were rescued with an *Nnt *transgene which improved the insulin secretion [34]. Furthermore,*in vitro *analyses using insulin-secreting Min6 cells and isolated islets with reduced *Nnt *function demonstrated diminished insulin secretion associated with lower ATP levels [35]. An intact *Nnt *gene might be expected to lead to higher ATP levels in BLKS β cells and thus increased insulin secretion.

**Figure 4:**
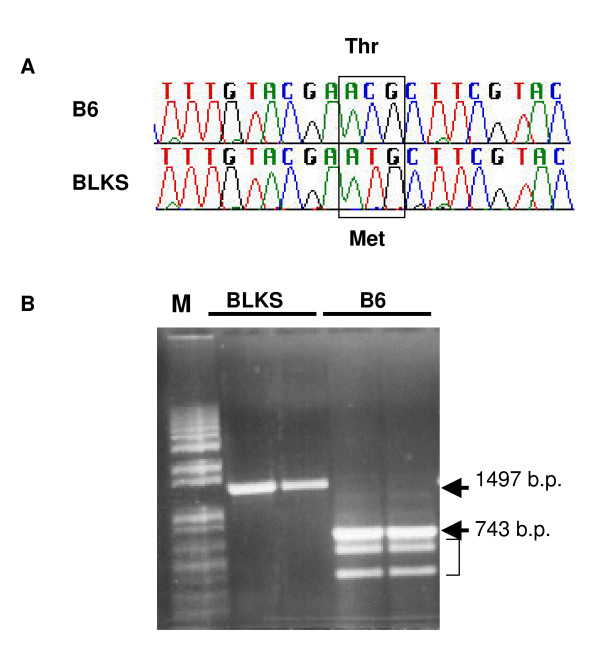
**Nicotinamide nucleotide transhydrogenase is derived from the DBA strain in BLKS mice**. (A) Sequencing of exon 1 of the *Nnt *gene in BLKS and B6 mice confirms the DBA origin of the gene in the BLKS genome. B6 mice possess the codon for threonine (Thr) at residue 35, whereas the BLKS mice have the codon for methionine (Met). (B) RT-PCR analysis of the *Nnt *RNA in B6 and BLKS. A smaller RT-PCR fragment is amplified from B6 RNA (743 bp) than BLKS RNA (1798 bp), confirming the deletion of exons 8 to 11 previously described in B6 mice [32]. The two smaller bands (bracket) in B6 mice are likely mis-splicing artifacts from downstream exons which are flanked by the PCR primers. M – marker.

To investigate the origin of the *Nnt *gene in BLKS mice, we first investigated its genomic location at 116.1 Mb on chromosome 13 and found that this locus was predicted to be derived from the DBA parental genome [9], suggesting that the gene in BLKS mice was intact. Next, we sequenced the first coding exon, previously reported to contain a point mutation in B6 mice [32]. In DBA mice, codon 35 reads ATG, resulting in methionine, whereas in B6 mice this codon is ACG, resulting in threonine. We found that the DBA strain contributed this exon of the *Nnt *gene in BLKS mice due to the presence of the ATG at codon 35 (Figure 4A). B6 mice also have a deletion of exons 8 to 11 in this gene [32]. To confirm that the BLKS mice did not carry this multi-exon deletion we performed RT-PCR on islet RNA with primers spanning the deletion (Figure 4B). Corresponding to the intact gene from DBA mice, a 1497 bp amplicon was generated from the DNA of BLKS mice, whereas in B6 mice a smaller 743 bp fragment was observed, as expected, due the deletion. We also observed two smaller fragments of 402 and 598 bp in B6, and inspection of the gene sequence suggests that these fragments are likely a result of the mis-splicing of exon 7 to exons 13 and 14. Altogether, these data show that *Nnt *is contributed by the DBA strain in BLKS and is intact, in contrast to the disrupted B6 gene. Thus, the higher islet expression of *Nnt *in BLKS mice relative to the B6 mice is most likely due to the lack of mutation in the DBA-derived allele, and may lead to higher ATP levels and higher insulin secretion, accounting for the enhanced glucose tolerance. Recently a study was published identifying a strong QTL on chromosome 13 governing insulin hypersecretion in a B6 × DBA cross [36]. *Nnt *was identified as the likely candidate gene and higher gene expression and intact gene structure was observed in islets. These data support our results and confirm the fact that *Nnt *gene is inherited from the DBA genome in BLKS mice.

The observations of altered expression of *Plagl1 *and *Nnt *between these two strains could account for the phenotypic differences observed in insulin-resistant states, such as enforced by deficiency of leptin or its receptor. Their individual prior association with human and/or mouse diabetes suggest these genes are functionally relevant to the proper action or survival of the β cell. The expression differences of these two genes alone may possibly be sufficient to elicit a physiological effect through their interaction. Indeed, the elevated secretion of insulin expected in β cells with intact *Nnt *function may precipitate an apoptotic event in a pro-apoptotic, *Plagl1*-enriched cell.

### Expression differences are mainly contributed by the DBA loci

We hypothesized that the DBA genome contributes to the islet dysfunction. The locations of the DBA blocks in the BLKS genome have been mapped by other groups [9-11]. We noticed that *Plagl1 *and *Nnt *were located in DBA loci of the BLKS genome, and investigated the genetic origin of the other genes in Table 2. We found that 28 out of these 32 genes were derived from DBA loci (Table 2, last three columns), based on the genetic mapping of Davis et al [9].

**Figure 5:**
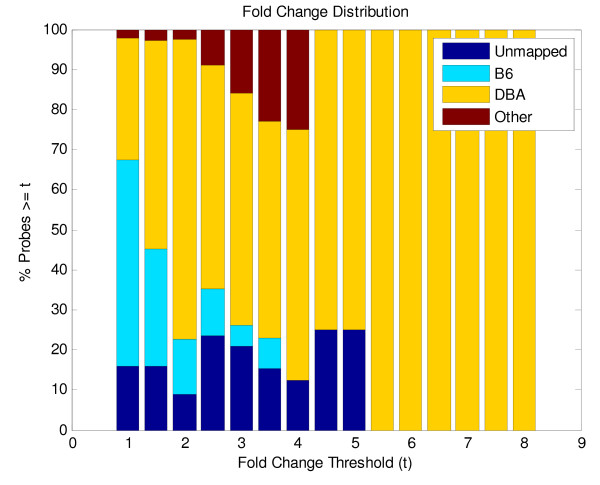
**Gene expression differences in BLKS islets demonstrate a major contribution by DBA located probes**. Gene expression fold-change differences for all probes in the experiment were assigned to either B6, DBA or *Other *genomes based on chromosomal location. The percentage of probes greater than the fold change threshold (*t*) are plotted against *t *for each set of genomically assigned probes. The contributions at a threshold of 1 reflect the baseline genomic contribution of these probes. As the fold change threshold increases, the B6 differences sharply decline, whereas the DBA probes increase their contribution. Above a fold change of 4.5, all of the expression differences are accounted for by DBA assigned probes.

We extended these observations of genomic location and expression fold change to the entire genome. Using the published mapping data [9] we identified the B6, DBA, and *Other *loci. *Other *loci were informative regions, but not classifiable as either B6 or DBA. Within these loci we mapped all the probes used in the profiling experiment and combined these data (Additional file 1, supplemental figure). All of the probes were segregated into genome sets, based on location of parental genomes (as described for Additional file 1, supplemental figure). Not all probes could be mapped and were labeled as *unmapped*, due to the inability to assign probes in intervals between markers derived from different strains. Figure 5 shows the fold changes of the probe sets from the B6, DBA and *Other *genomes. Microarray probes appear to be uniformly distributed across the mouse genome, and among the haplotype blocks. As the threshold increases, the contribution of the B6 genome declines, and no differences above a threshold of 3.5 were detected (Figure 5). In contrast to the B6 contribution, the contribution of the DBA genome increases as the fold change cut-off increases. At a 2-fold change or greater cut-off, we observed that 75% of the probes were DBA in origin, representing 10 times as many B6 probes. Furthermore, these data show a contribution of the *Other *genome within the range of 2 to 4-fold; up to 23% of the probes with a fold change greater than or equal to four are from genes located in the *Other *genome.

We used the Kruskal-Wallis test to determine if there was a statistical difference in global gene expression fold-change distributions between probes from the different genomes. At the level of the whole genome, we found there was very high statistical significance for fold-change differences between DBA, B6 and *Other *(χ^2 ^= 126.52, *P *< 4× 10^-27^, for probes with significant [*P *< 1× 10^-6^] fold changes). We also investigated each chromosome individually using the same test, and found that many chromosomes showed significant differences in fold change between block types (Additional file 1, supplemental table). Chromosomes 6, 7, 8, 13 and 17 were all significant for fold-change distribution differences between the genomes. These data indicate an association of the DBA loci with the gene expression differences in BLKS mouse islets. In a QTL screen to identify loci which influence metabolic phenotypes between B6 and BLKS mice, Mu and colleagues [37] identified two suggestive LOD scores for plasma glucose on chromosomes 8 and 17, both identified in our study to harbor significant gene expression differences. Additionally, the recent report by Aston-Mourney and co-workers [36] identified suggestive LOD scores for insulin secretion on chromosomes 5, 6 and 7, of which two were identified in our study.

## Conclusion

We have analyzed the physiological differences between B6 and BLKS mice with attention to their glucose homeostasis/islet phenotype. We have shown several transcriptional differences linked to diabetes and inflammatory mechanisms leading to islet damage. Glucose stimulation appears to elicit inflammatory responses, and particularly increases in the genes *Lcn2*, *Spp1*, *S100a9*, and *Slc7a3*. These changes are seen in both strains, but expression is always higher in BLKS mice. Altogether this suggests that islet dysfunction in BLKS has a strong inflammatory component. The largest islet gene expression differences were in *Plagl1 *and *Nnt*, two genes implicated in β-cell biology. We identified genetic differences in these genes between the two strains. The interaction of these two genes may account for the β-cell dysfunction commonly observed in BLKS mice. Lastly, we undertook a genomic approach to translate islet expression data, and revealed a significant and primary contribution from the DBA genome. These data demonstrate the utility of combining genomic, genetic, and physiological data to aid in the delineation of multi-factorial phenotypes.

## Methods

### Animal studies

All mice were maintained in accordance with the Institutional Animal Use and Care Committee of Amgen Inc. B6 and BLKS male mice were obtained at 6 weeks of age from The Jackson Laboratory (Bar Harbor, ME). Mice received food and water *ad libitum *and were maintained on a 12:12 h light-dark cycle (lights on at 6:30 am) and housed one per cage. Fasting (4 h) blood samples (150 μl) were collected from the retro-orbital sinus of non-anesthetized mice into EDTA plasma tubes and blood glucose was measured using a OneTouch Profile glucometer (LifeScan, Milpitas, CA). For GTT, mice were fasted from 9:00 pm to 9:00 am. Blood glucose was measured at 9:00 am, and an intraperitoneal glucose bolus (2 g/kg body weight) was administered to conscious, unrestrained mice. During the GTT, blood glucose was measured at 30, 60 and 120 min. Plasma insulin levels were analyzed using the LINCOplex mouse endocrine immunoassay panel following the manufacturer's instructions (Millipore, St. Charles, MO).

### Pancreatic islet isolation

Pancreatic islets were isolated from 12-week-old mice. After clamping the common bile duct as it joins the intestine, the pancreas was inflated with 5 ml of collagenase type XI (0.6 mg/ml; Sigma-Aldrich, St. Louis, MO) diluted in Hanks balanced salt solution (HBSS; Sigma-Aldrich). The distended pancreas was removed and incubated at 37°C for 10 min, and the islets were dispersed by gentle shaking. Enzymatic digestion was stopped by the addition of 45 ml of cold HBSS containing 10%FBS. Two rounds of centrifugation (300× g for 2 min) and washes with fresh HBSS/FBS were carried out, and the islets were resuspended in 10 ml of HBSS/FBS buffer. The slurry was layered on top of a prepared histopaque gradient (comprised of a 10 ml lower layer of Histopaque 1.119, and 10 ml upper layer of Histopaque 1.077; Sigma-Aldrich). Following centrifugation of the gradient at 1000× g for 30 min, the islets were collected from the top of the 1.077 interface, pipetted onto a 70 μm cell strainer, and washed with HBSS/FBS buffer. Finally, the islets were rinsed into a Petri dish, isolated from any contaminating exocrine material visually using a dissecting microscope, and cultured in RPMI-1640 media (Cellgro, University of Miami, FL), supplemented with 10%FBS and 100 U/ml of penicillin, and 100 μg/ml of streptomycin at 37°C in 5% CO_2_. Prior to all experiments, islets were cultured for 48 h in RPMI-1640 media containing 11 mM glucose, and reselected for use based on morphology as assessed with a dissection microscope.

### Glucose-stimulated insulin secretion assay

Twenty-four hours prior to the secretion assay, islets were picked into 24-well inserts (Multiwell Insert System, 8.0 μm pore size; BD Biosciences, San Jose, CA) in groups of 10 similarly sized islets and cultured in 1 ml/well RPMI-1640. For the insulin secretion assay, Krebs-Ringer Bicarbonate (KRBH) Buffer was prepared (129 mM NaCl, 4.8 mM KCl, 1.2 mM KH_2_PO_4_, 1.2 mM MgSO_4_.7H_2_O, 10 mM HEPES, 2.5 mM CaCl_2_.2H_2_O and 4.8 mM NaHCO_3_) and oxygenated for 15 min with 95%O_2_/5%CO_2_. Bovine serum albumin (fatty acid-free, Cat. No. A6003; Sigma-Aldrich) at a final concentration of 0.625% was added to the buffer, then the buffer was warmed to 37°C and pH adjusted to 7.4 with 5 M NaOH. The islets were starved for one hour at 37°C in KRBH + 1 mM glucose (Cat. No. 99-787-CI, Cellgro) by transferring the 24-well insert from the RPMI-1640 culture media to a new 24-well plate containing 1 ml/well KRBH + BSA and 1 mM glucose. The islets were transferred to the experimental plates containing 1 ml/well of KRBH + BSA and 1 mM, 10 mM and 16.7 mM glucose. Following a one-hour incubation at 37°C, the insert containing the islets was removed and the buffer plate frozen at -80°C. The insulin ELISA was carried out using the Ultra Sensitive Mouse Insulin ELISA kit (Cat. No. 90080; Crystal Chem Inc. Downers Grove, IL). Five microliters of each buffer sample was run in duplicate per assay protocol.

### DNA and RT-PCR analysis

DNA was isolated from tail tissue and used for PCR and direct sequencing analysis of the *Nnt *gene. RT-PCR using the One-Step System (Invitrogen, Carlsbad, CA) and islet RNA was performed using the oligonucleotides, Nnt forward-TACAAGAGCTGCCGCTTTGGA, and Nnt reverse-AGACCCACTAAAGGTGACTCCG; the product was used for direct DNA sequencing.

#### qRT-PCR

RNA isolation kits from QIAGEN (micro-RNAeasy kit; Valencia, CA) were used to prepare RNA from purified islets with a final eluate of 15 μl per mouse. RNA was quantified using Agilent's 2100 Bioanalyzer (Santa Clara, CA). Real time PCR was performed using 0.2 μM of *Plagl1 *primer and probe (forward-TCAAGTGCTCGAAGGCTGAGT, reverse-TGTGTGGCCATGTGTCTCATC, probe-FAM-TGGCAAAGCCTTCGTCTCCAAGTATAAGC-BHQ). Primers were derived from different exons to avoid amplification of any residual genomic DNA. The RT-PCR reactions were done using QIAGEN's Quantitect Multiplex RT-PCR kit. The total reaction volume was 20 μl per well and 20 ng RNA per reaction. Eight replicates of islets isolated from B6 and four replicates of BLKS were run with *cyclophilin *used as the housekeeping gene. Amplification and quantification of PCR products was performed on an ABI Prism 7900 (Applied Biosystems, Foster City, CA). Data were exported into Excel and analyzed using the delta CT method.

### Islet histology

Pancreata were removed and fixed in buffered zinc formalin and embedded in paraffin. Pancreas sections (5 μm) were deparaffinzed, hydrated and blocked for non-specific reactivity with CAS block (Zymed Lab., San Francisco, CA). Sections were incubated with guinea pig anti-swine insulin (A562; DAKO, Carpinteria, CA) at 1:3000 for 45 min at room temperature. Insulin was detected by biotinylated goat anti-guinea pig (BA-7000; Vector Laboratories Inc., Burlingame CA) at 1:100. Slides were quenched with 3% H_2_O_2 _and followed with avidin-biotin HRP Complex (Vector Lab., Burlingame CA). Reaction sites were visualized with DAB (DAKO). Next, slides were rinsed with PBS thoroughly and incubated with rabbit anti-human glucagon (A565; DAKO) at 1:800 for 1 h at room temperature. Glucagon was detected by a biotinylated goat anti-rabbit secondary (BA-1000; Vector Lab.) at 1:200 and followed with avidin-biotin alkaline phosphatase complex. Reaction sites were visualized with alkaline phosphatase substrate (Vector Lab.).

### Experimental design for islet expression profiling

We investigated gene expression differences in islets from B6 and BLKS mice and between pretreatments in the presence of low (5 mM) and high glucose (20 mM) in the culture media (to simulate basal and stimulated conditions respectively). To provide >85% confidence that each fold change >1.5 was true, we used *n *= 5 per group. To eliminate differences in gene expression contributed by variation across mice within a group, we did not pool islets from different animals but kept them separate. Islets were then cultured separately for 24 h in RPMI-1640 media containing either 5 mM or 20 mM glucose. At the end of the culture pretreatment, the islets were picked from the culture vessel, rinsed briefly in HBSS/FBS buffer and homogenized in RLT buffer. RNA was prepared using the micro-RNAeasy kit (QIAGEN). On average, 580 ng of total RNA was recovered from the isolated islets from each individual mouse.

### Pancreatic insulin content

Pancreata were excised and frozen in liquid nitrogen and stored at -80°C. Protein content was determined using the BCA Protein Assay Kit (Thermo Scientific Rockford, IL) following homogenization of the pancreata for 2 min at 25 Hz in 1 ml of 0.18 M HCl and 70% ETOH in a TissueLyser (Qiagen Gmbh, Germany). The tissue lysate was clarified for 10 min at 4000 rpm and the supernatant was transferred to a fresh microcentrifuge tube. For the protein assay, the supernatant was 10× diluted in PBS. The insulin content assay was performed using Ultra Sensitive Mouse Insulin ELISA kit (Crystal Chem). The tissue lysate was diluted 1000× and 6000× with sample diluent supplied with the kit before running the assay. All samples for both assays were run in duplicate.

### Expression profiling

Total RNA was profiled following the Agilent Two-Color Microarray-Based Gene Expression Analysis Protocol v4.0.2. Briefly, 200 ng of total RNA from each sample was separately amplified and labeled with Cy3- and Cy5-labeled CTP (Perkin Elmer; Wellesley, MA) using the Agilent low-input linear amplification kit. Labeled cRNA was purified using the Qiagen RNeasy Mini kit protocol for liquid samples (QIAGEN). Purified cRNA was quantified using the Nanodrop Fluorospectrometer ND-3000 (Wilmington, DE). Similar amounts of Cy3- and Cy5-labeled cRNA were combined and fragmented for 30 min at 60°C. The products were hybridized to Agilent Mouse Whole Genome Microarrays, and the resulting array data were extracted using Agilent Feature Extraction Software v8.1.

### Bioinformatic analysis

Raw data for each array were analyzed with Matlab software (The Mathworks Inc. Natick, MA). Data were not analyzed as dye ratios, but as dye-normalized relative fluorescence units (RFUs). Dye normalization was accomplished by pairing Cy3 and Cy5 values, and determining the effect of dye on RFU magnitude by including dye as a factor in an ANOVA analysis of the binary logarithm-transformed probe intensities, including all two-factor interactions. The other factors in the ANOVA were strain and glucose pretreatment. Found to be significant, the effect of reporter dye was subtracted in a least-squares manner. Ultimately, technical and then biological replicates were averaged prior to fold-change calculations. Raw and normalized data discussed in this publication have been deposited in National Center for Biotechnology Information's Gene Expression Omnibus (GEO, ) and are accessible through GEO Series accession number GSE11257.

Genotyping data from Davis et al [9] were used to divide the genome into DBA and B6 regions. Each region is bordered by two markers for the same strain with no intervening markers of any other strain. Regions with informative SNPs, not attributable to DBA or B6 were labeled *Other*. Each microarray probe was mapped to a region by determining if its target gene overlapped with any identified region. Probes not mapped to one of the three regions above were labeled as *Unmapped*. Fold-change distributions of the four classes of probe were compared using the Kruskal-Wallis test, a nonparametric ANOVA testing the hypothesis that groups have the same median fold-change rank. Fold changes were calculated between BLKS and B6 mice under high-glucose conditions, and those with a strain effect *P *value of < 1 *E*-6 were used in the test. This test, for differences between regions, was executed for the entire genome and for each chromosome individually.

## Competing interests

The authors declare that they have no competing interests.

## Authors' contributions

AAA carried out the gene expression data analysis, the SNP analysis, and participated in drafting the manuscript. JH did the glucose tolerance testing, the insulin ELISA for islet insulin secretion experiment, the measurement of pancreatic insulin content, the *Plagl1 *qPCR, and the *Nnt *RT-PCR. TJ and CL performed the sequencing and related analysis. DJL prepared RNA from islets isolated by JM, MG, and DJL. DMB and MAD carried out the expression profiling. MMV participated in drafting the paper. DJL conceived of the study, participated in its design and coordination, performed experiments and their analysis, and participated in drafting the manuscript. All authors read and approved the final manuscript.

## Supplementary Material

Additional file 1 **Supplementary data.** Figure showing location of the probes from the islet expression profiling compared to the genomic location of the contributing genomes. Table showing differences in gene expression between genomic regions (DBA, B6, *Other*, Unmapped) at the whole genome and chromosomal levels. Click here for file
