# Supplementary material for: Pancreatic islet expression profiling in diabetes-prone C57BLKS/J mice reveals transcriptional differences contributed by DBA loci, including Plagl1 and Nnt
Source: Pathogenetics. 2009 Jan 22;2:1. doi: 10.1186/1755-8417-2-1 (PMC2642818; doi:10.1186/1755-8417-2-1)
Supplement: Additional file 1 — Supplementary data. Figure showing location of the probes from the islet expression profiling compared to the genomic location of the contributing genomes. Table showing differences in gene expression between genomic regions (DBA, B6, Other, Unmapped) at the whole genome and chromosomal levels. [file 1755-8417-2-1-S1.doc]

**ANDERSON ET AL: SUPPLEMENTAL DATA**

**Supplemental Figure 1: Location of the probes from the islet expression profiling compared to the genomic location of the contributing genomes.** Each spot represents a different Agilent probe, and is graphed on the x-axis to reflect the absolute log ratio of the difference in expression between the two strains. The y-axis indicates, from top-to-bottom, the location along each chromosome as identified from the mapping data of Davis et al. [1]. Red spots indicate those probes with a log ratio greater or equal to 1.5. The genomic composition is shown of the right hand side of each chromosomal plot, and illustrates the contributing genomes B6 (blue), DBA (orange) or other (gray). A locus which is not colored was not assigned to any of the three genomes due to insufficient SNP data to locate the precise end of one genome and the start of the next genome.

**Chromosome**

1. Davis RC, Schadt EE, Cervino AC, Peterfy M, Lusis AJ: **Ultrafine mapping of SNPs from mouse strains C57BL/6J, DBA/2J, and C57BLKS/J for loci contributing to diabetes and atherosclerosis susceptibility.** *Diabetes* 2005, **54:**1191-1199.

**Supplemental Table 1.** **Differences in gene expression between genomic regions (DBA, B6, *Other*, Unmapped) at the whole genome and chromosomal levels**. Expected fold change (*P <* 1E-6) for each genomic region expressed as a mean with medians in parentheses. *Whole Genome. **NaN indicates that a region has no significant probes represented. P-value significance from the Kruskal-Wallis test.

| **Chromosome** | **DBA** | | **B6** | | **Other** | | **Unmapped** | | **P-value°** |
| --- | --- | --- | --- | --- | --- | --- | --- | --- | --- |
| **1** | 1.41 | (1.37) | 1.46 | (1.36) | NaN** | NaN | 1.47 | (1.47) | 8.54E-01 |
| **2** | NaN | NaN | 1.36 | (1.32) | NaN | NaN | NaN | NaN | 1.00E+00 |
| **3** | 1.50 | (1.35) | 1.44 | (1.37) | NaN | NaN | 1.81 | (1.53) | 3.72E-01 |
| **4** | 1.50 | (1.35) | 1.52 | (1.41) | NaN | NaN | 1.76 | (1.58) | 2.05E-01 |
| **5** | 1.60 | (1.49) | 1.54 | (1.45) | 1.61 | (1.61) | 1.59 | (1.39) | 9.42E-01 |
| **6** | 2.00 | (2.06) | 1.20 | (1.12) | NaN | NaN | 1.51 | (1.45) | **0.00E+00** |
| **7** | 2.11 | (1.67) | 1.48 | (1.32) | NaN | NaN | NaN | NaN | **2.20E-02** |
| **8** | 1.74 | (1.58) | 1.30 | (1.27) | 1.63 | (1.63) | 1.50 | (1.46) | **4.07E-02** |
| **9** | 1.47 | (1.39) | 1.42 | (1.35) | 1.35 | (1.35) | 1.70 | (1.31) | 9.55E-01 |
| **10** | 2.30 | (1.44) | 1.42 | (1.33) | NaN | NaN | 1.19 | (1.19) | 1.82E-01 |
| **11** | 2.66 | (1.59) | 1.73 | (1.71) | 1.79 | (1.62) | 1.42 | (1.49) | 3.33E-01 |
| **12** | 1.56 | (1.44) | 1.41 | (1.41) | NaN | NaN | 1.29 | (1.29) | 6.43E-01 |
| **13** | 1.68 | (1.68) | 1.50 | (1.51) | 4.05 | (4.07) | 2.13 | (1.95) | **2.25E-02** |
| **14** | 1.82 | (1.68) | 1.46 | (1.47) | NaN | NaN | 1.67 | (1.67) | 2.39E-01 |
| **15** | 1.60 | (1.56) | 1.60 | (1.65) | 1.51 | (1.51) | 1.64 | (1.59) | 9.20E-01 |
| **16** | 1.70 | (1.77) | 1.21 | (1.21) | 1.18 | (1.18) | 1.96 | (1.57) | 2.08E-01 |
| **17** | 1.87 | (1.6) | 1.29 | (1.29) | NaN | NaN | NaN | NaN | **2.98E-02** |
| **18** | 1.41 | (1.31) | 1.51 | (1.45) | NaN | NaN | 1.46 | (1.46) | 9.85E-01 |
| **19** | 1.41 | (1.32) | 1.43 | (1.37) | NaN | NaN | 1.76 | (1.58) | 1.83E-01 |
| **X** | 1.66 | (1.39) | 1.32 | (1.32) | NaN | NaN | 1.61 | (1.4) | 7.32E-01 |
| **WG*** | 1.84 | (1.72) | 1.42 | (1.33) | 2.05 | (1.62) | 1.67 | (1.51) | **0.00E+00** |
